# Warm autoimmune hemolytic anemia in adults in Latin America: A scoping review

**DOI:** 10.1016/j.htct.2026.106351

**Published:** 2026-03-08

**Authors:** Juan Antonio Flores-Jiménez, Sandra Fatima Menosi Gualandro, Diogo Ribeiro, Yudy Medina, Renato Watanabe de Oliveira, Pamella Villanova

**Affiliations:** a Centro Universitario de Tonalá, Universidad de Guadalajara, Guadalajara 45425, Mexico; b Department of Hematology, Faculdade de Medicina, Universidade de São Paulo, São Paulo 05508-220, Brazil; c CTI Clinical Trial & Consulting Services, 1070-274 Lisboa, Portugal; d Market Access, Pricing & HEOR Center of Excellence LATAM, Johnson & Johnson, Bogotá DC 111071, Colombia; e RWE LATAM, Johnson & Johnson, São Paulo, SP CEP 04543-011, Brazil; f Immunology LATAM, Johnson & Johnson, Av. Presidente Juscelino Kubitschek, 2041- 8°floor/N8. Complexo JK - Bloco B, São Paulo, SP CEP 04543-011, Brazil

**Keywords:** Autoimmune hemolytic anemia, Latin America, Adult, Review, Treatment outcome

## Abstract

**Background:**

Autoimmune hemolytic anemia is a rare disorder characterized by the autoimmune-mediated destruction of red blood cells. The condition is classified into subtypes based on the thermal reactivity of the autoantibodies, with warm autoimmune hemolytic anemia representing the most common form in adults. This review aimed to summarize the clinical evidence regarding warm autoimmune hemolytic anemia generated in adult patients within the Latin American region.

**Methods:**

A literature search was conducted in Embase, Medline, Lilacs, Cochrane Library, Epistemonikos, and Value in Health in April 2023. Gray literature was also consulted. Records were eligible for inclusion if they presented data on the epidemiology, diagnosis, treatment, or healthcare resource utilization among adult warm autoimmune hemolytic anemia patients in Latin American countries. Clinical practice guidelines on the condition issued in Latin American were also eligible.

**Results:**

Nine records were included: seven retrospective studies (six single-center), one national clinical guidelines (Mexico), and one therapeutic protocol (Brazil). Corticosteroids are the cornerstone of first-line treatment, reported in 97.7 % of 354 patients enrolled in the studies, primarily as monotherapy (91 %). While response rates were high (69–84 %), a considerable proportion of patients (15–40 %) required second-line therapies. The use of splenectomy varied widely between studies, from being the preferred second-line option to not being performed in any patient. Access barriers contribute to a limited use of rituximab.

**Conclusion:**

Compared to other hemolytic disorders, warm autoimmune hemolytic anemia studies are lacking in Latin American and consist primarily of single-center, retrospective case series. Further research is warranted to understand management strategies in refractory disease.

## Introduction

Autoimmune hemolytic anemia (AIHA) is a rare disorder characterized by an autoimmune-mediated destruction of red blood cells (RBCs) [1]. Its epidemiology is not well described, but a study in Denmark estimated 1.77 new cases per 100.000 person-years from 2008 to 2016 [2]. Despite being a rare disease, AIHA has a relevant impact on both patients and society and is associated with important healthcare resource utilization, namely due to highly expensive drugs, hospital admissions, and frequent outpatient visits and blood transfusions [3].

The clinical picture in AIHA varies widely, ranging from no symptoms to severe life-threatening hemolysis [4]. AIHA is classified according to the temperature reactivity of the pathogenic antibodies as warm AIHA (wAIHA), cold AIHA, or mixed AIHA [5]. wAIHA, in which autoantibodies target RBC surface antigens at body temperature and trigger hemolysis [6], accounts for the majority of all AIHA cases in the adult population (60–70 %) [1,7]. wAIHA is further categorized as primary (idiopathic) or secondary depending on whether there is an underlying cause. Approximately half of all wAIHA cases are attributed to lymphoproliferative neoplasms, autoimmune and infectious diseases, immunodeficiencies, solid tumors, transplants, or drugs [1,7]. Thrombotic events and infections secondary to immunosuppressive interventions are known complications of wAIHA and can potentially be fatal [[8], [9], [10]].

The direct antiglobulin test (DAT) is the keystone of AIHA diagnosis with its findings being critical to distinguish between the various subtypes [[11], [12], [13]]. In wAIHA, the DAT is typically positive for immunoglobulin (Ig)G only, but it may also show positivity for both IgG and complement component 3d (C3d) or, less commonly, C3d alone. Cold agglutinin disease is characterized by DAT positivity for C3d only. Mixed forms of AIHA demonstrate DAT positivity for both IgG and C3d, with coexistence of warm autoantibodies and a high titer of cold agglutinins. Patients may also present with DAT-negative AIHA, in which testing fails to detect IgG on RBCs despite ongoing hemolysis. This is most commonly due to levels of RBC-bound IgG falling below the detection threshold of standard DAT methods. Less frequently, it may result from low-affinity IgG or from RBC-bound immunoglobulins of other classes, such as IgA or, more rarely, IgM [13].

DAT-negative AIHA occurs in 5–10 % of patients with unequivocal evidence of the disease [7,13]. Consequently, a negative DAT result is insufficient to exclude a diagnosis of wAIHA [7]. The diagnosis is supported by a combination of clinical symptoms, including fatigue, syncope, chest pain, weakness, and exertional dyspnea, and laboratory evidence of hemolysis, specifically anemia, jaundice, and splenomegaly. Characteristic biochemical findings include elevated indirect bilirubin, increased lactate dehydrogenase (LDH), reticulocytosis, and depleted serum haptoglobin levels [5,[14], [15], [16]].

The goal of wAIHA treatment is to reduce the degree of hemolysis, consequentially increasing hemoglobin (Hb) levels and improving symptoms. Management of the underlying cause is crucial in secondary wAIHA, either by treating the associated lymphoproliferative or autoimmune disease or by stopping the drugs that might be causing the disease [17]. Evidence on the treatments used for wAIHA is limited, with a scarcity of randomized prospective clinical trials. As a result, treatment has historically been based on expert opinions and retrospective studies [[18], [19], [20], [21], [22]].

Corticosteroids are the mainstay first-line treatment for wAIHA, with a response rate of 70–80 % [23,24]. However, between 20 and 35 % of patients fail to respond to corticosteroid treatment or require excessively high maintenance doses to control hemolysis [25]. Published relapse rates in wAIHA after first-line corticosteroid therapy vary widely, ranging from 10.5– 68 % among patients who initially respond to treatment [[26], [27], [28]]. In such cases, second-line treatments are employed, mainly splenectomy, rituximab, and immunosuppressants.

The present review aimed to evaluate and summarize the available evidence on adult patients with wAIHA in Latin America. Specifically, this study intended to assess the epidemiology, patient journey (diagnosis, treatments, and outcomes), clinical burden (morbidity and mortality), and healthcare resource utilization among patients with wAIHA, as well as to identify any unmet needs in the management of this condition.

## Materials and methods

This scoping review is reported in accordance with the Preferred Reporting Items for Systematic Reviews and Meta-Analyses extension for Scoping Reviews (PRISMA-ScR) [29].

### Literature search

A literature search was carried out in Embase, Medline, Lilacs, Cochrane Library, Epistemonikos, and Value in Health in April 2023. The search terms aimed at retrieving literature focused on wAIHA in Latin America. Detailed information on the search strategy is provided in Supplementary Table 1. Gray literature was also consulted. The reference lists of selected articles were searched for additional records.

### Selection criteria

Studies were eligible for inclusion in the present literature review if: 1) data were provided on the epidemiology, diagnosis, treatment, or healthcare resource utilization of patients with wAIHA and 2) results were available for adult patients (≥18 years) in Latin American countries. Review articles, case reports, letters to editor, and commentaries were excluded. Gray literature was eligible if published within the last ten years. This period was extended to 15 years for any other publication. Any clinical practice guidelines on wAIHA issued in Latin America was eligible for inclusion in the review. Only reports in English, Portuguese, or Spanish were considered.

The Rayyan platform was used to screen the titles and abstracts of records retrieved from the literature search. The full-text versions of all those complying with the pre-established criteria were obtained and reviewed for eligibility. Records that did not meet with the selection criteria were excluded. Two reviewers selected records for inclusion, with disagreements settled by a third reviewer.

### Data extraction process

Data were extracted from studies into an electronic data extraction form. These data included the primary author, year of publication, country, study type, number and characteristics of enrolled wAIHA patients, treatments for wAIHA, and reported outcomes.

## Results

### Study selection

**Figure 1:**
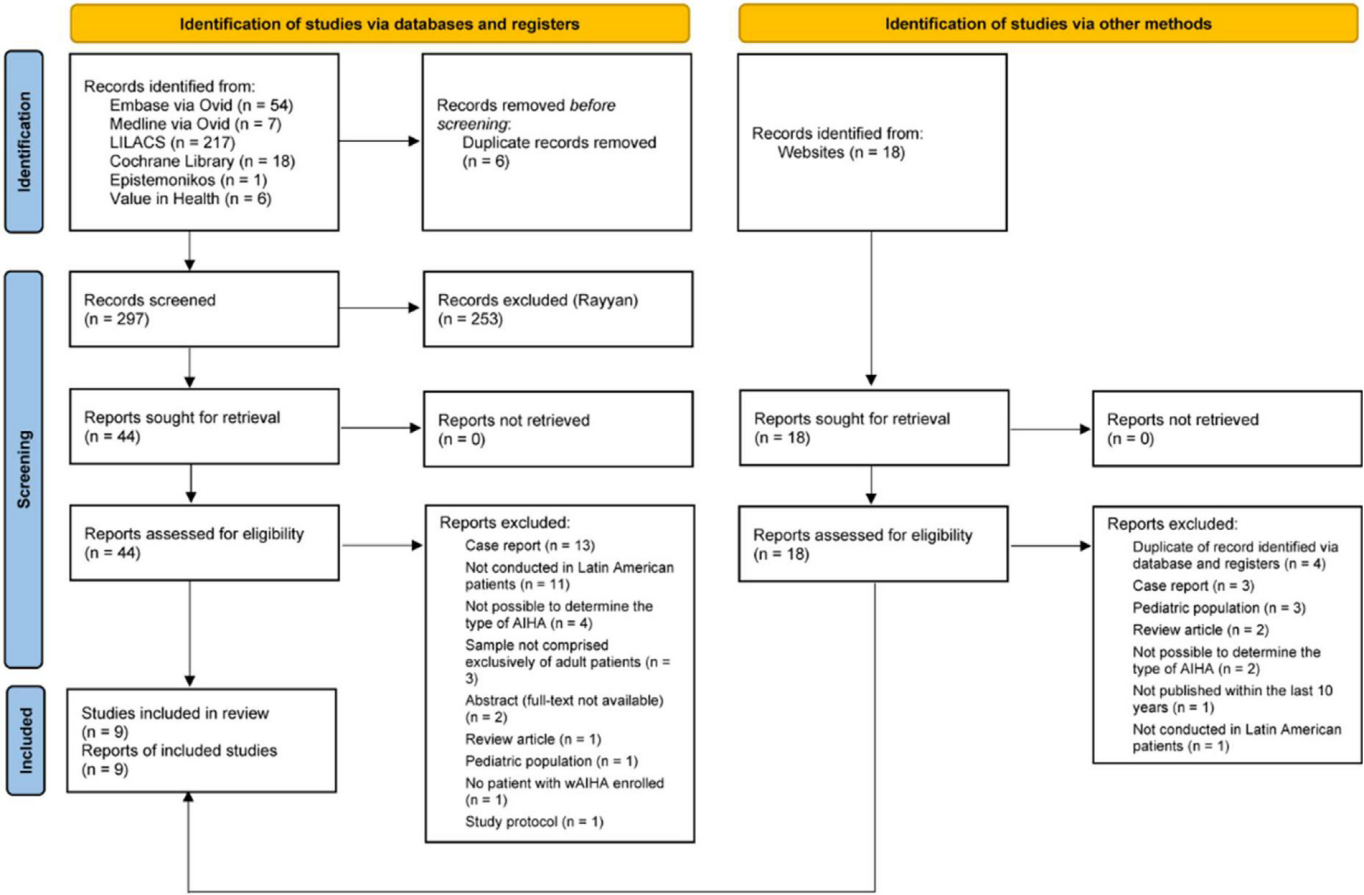
Flow diagram of literature search. Modified from the Preferred Reporting Items for Systematic Reviews and Meta-Analysis 2020 flow diagram for new systematic reviews.

The literature search retrieved a total of 321 records (Figure 1), of which 303 were identified via databases and registers and 18 from gray literature. Six records were duplicates and were removed. Forty-four of the records identified using the database search potentially met eligibility criteria following screening of the titles and abstracts using Rayyan. The review of the full-text versions led to the exclusion of 37 records, resulting in seven eligible records. Two of the 18 records retrieved from gray literature were also eligible, giving a total of nine records included in the present review. Overall, the included records consisted of seven retrospective studies, one national clinical guidelines, and one therapeutic protocol.

### Characteristics of included studies

Overall, seven retrospective analyses of series of patients treated for wAIHA in Latin American countries (three in Cuba, two in Mexico, one in Chile, and one in Colombia) were included. Of these, six were single-center studies. No randomized clinical trials met the eligibility criteria.

A total of 354 wAIHA patients were enrolled in these studies. The characteristics of the studies and enrolled populations are summarized in Table 1.

**Table 1:** Summary of included studies.

Author, Year	Country	Study type	Date of wAIHA diagnosis	Patient characteristics	DAT positivity and immunoglobulin pattern	Secondary wAIHA	Clinical presentation
Jaime-Pérez et al. 2019 [14]	Mexico	Single-site, retrospective, cohort study	2002 - 2017	64 patients 60.9 % female Median age at diagnosis: 37 (range: 16 to 77 years)	100 % DAT positivity^a^	33 patients (51.6 %) • Systemic lupus erythematosus (21.9 %) • Evans syndrome (18.8 %) • Antiphospholipid syndrome (3.1 %) • Hyperthyroidism (3.1 %) • Chronic lymphocytic leukemia (1.6 %) • Hodgkin lymphoma (1.6 %) • Rheumatoid arthritis (1.6 %)	Anemic syndrome (100 %)^b^
López-Vidal et al. 2019 [31]	Chile	Single-site, retrospective, cohort study	2010–2018	36 patients 72 % female Mean age at diagnosis: 55 years (range: 22 to 81 years)	97.7 % DAT positivity	24 patients (66.7 %) • Lymphoid neoplasia (38.9 %) • Mesenchymopathy (16.7 %) • Other neoplasia (11.1 %)	No information on clinical presentation
Hernandez-Company et al. 2017 [5]	Mexico	Single-site, retrospective, cohort study	1992 - 2015	89 patients 73 % female Median age at diagnosis: 36 years (range: 17 to 86 years)	100 % DAT positivity^a^ Isolated IgG (71.9 %) IgG + C3d (28.1 %)	49 patients (55.1 %) • Systemic lupus erythematosus (34.7 %) • Primary antiphospholipid syndrome (30.6 %) • Hepatic disease (10.2 %) • Thyroid disease (6.1 %) • Solid malignancy (6.1 %) • Sjogren’s disease (2.0 %) • Psoriasis (2.0 %) • Non-Hodgkin lymphoma (2.0 %) • HIV infection (2.0 %) • Rheumatic fever (2.0 %) • Ingestion of allopurinol (2.0 %)	• Jaundice (78.7 %) • Anemic syndrome (71.9 %) • Splenomegaly (33.7 %) • Choluria (23.6 %)
Agramonte et al. 2015 [32]	Cuba	Single-site, retrospective, cohort study	2011–2013	15 patients 53 % female Median age at diagnosis: 59 years (range: 34 to 75 years)	93.3 % DAT positivity Isolated IgG (46.7 %) IgG + C3 (33.3 %) C3 (20.0 %)	2 patients (13.0 %) • Systemic lupus erythematosus (50.0 %) • Chronic lymphocytic leukemia (50.0 %)	• Jaundice (73 %) • Diffuse joint paint (20 %) • Splenomegaly (13 %) • Thrombotic events (13.3 %)
Quintero 2015 [30]	Colombia	Multi-site, retrospective, cohort study	2003–2013	71 patients 66.2 % female Median age at diagnosis (*n* = 70): 52 years	100 % DAT positivity^a^	35 patients (49.3 %) • Systemic lupus erythematosus (28.6 %)^c^ • Lymphoma (20.0 %) • Rheumatoid arthritis (8.6 %)^d^ • Connective tissue disorder (8.6 %) • Chronic lymphocytic leukemia (8.6 %) • Antiphospholipid antibody syndrome (5.7 %) • Myeloid neoplasms (5.7 %) • Infections (5.7 %) • Large granular lymphocyte leukemia (2.9 %) • Bronchogenic carcinoma (2.9 %) • Other (2.9 %)	No information on clinical presentation
Hernández et al. 2013 [34]	Cuba	Single-site, retrospective, cohort study	1997 - 2006	44 patients 72.7 % female Median age at diagnosis: 58 years (range: 27 to 73 years)	100 % DAT positivity Isolated IgG (63.6 %)^f^ IgG + C3 (36.4 %)^f^	0 %^e^	No information on clinical presentation
Valdés et al. 2009 [33]	Cuba	Single-site, retrospective, cohort study	Not reported	35 patients^g^ 65.7 % female Median age at diagnosis: 48 years (range: 18 to 60 years)	91.4 % DAT positivity IgG + C3 (62.5 %) Isolated IgG (18.7 %) C3 (16.6 %) IgA (3.1 %)	5 patients (16.6 %) • Systemic lupus erythematosus (40.0 %) • Mixed connective tissue disease (40.0 %) • Chronic lymphoproliferative syndrome (20.0 %)	No information of clinical presentation

wAIHA, warm autoimmune hemolytic anemia; DAT, direct antigen test.

a Inclusion in the study was limited to patients with a positive DAT per the eligibility criteria.

b No information on other symptoms was provided.

c One patient also presented antiphospholipid antibody syndrome.

d One patient also presented Sjogren’s disease.

e Inclusion in the study was limited to patients with idiopathic wAIHA per the eligibility criteria.

f Inclusion in the study was limited to patients with IgG antibodies per the eligibility criteria.

g Two of the 35 patients enrolled in the study had cold AIHA.

None of the included studies reported prevalence or incidence data for wAIHA in Latin American countries.

### Diagnosis

Inclusion was limited to patients with a positive DAT in three of the studies (Table 1) [5,14,30]. The rate of DAT positivity ranged from 91.4–100 % in the remaining four studies [[31], [32], [33], [34]]. In two of the three studies where at least one patient had a DAT-negative wAIHA result, enzyme immunoassays were employed to confirm the presence of immunoglobulins on the surface of RBCs [32,33]. The isolated IgG pattern was the most common in three of the four studies with available data, accounting for 46.7–71.9 % of patients [5,[32], [33], [34]].

### Sociodemographic characteristics and clinical presentation

wAIHA disproportionately affected women in the studies (Table 1), with all seven studies presenting study populations primarily composed of female patients (53–73 %). The median age at diagnosis ranged from 36–59 years.

Inclusion was limited to patients with primary wAIHA per the eligibility criteria in one of the studies [34]. Of the remaining six studies, the proportion of patients with secondary wAIHA varied from 13.0–66.7 %. Systemic lupus erythematosus was the most common underlying cause of wAIHA in five of these studies, accounting for 21.9–50.0 % of patients with secondary wAIHA [5,14,32,33]. López-Vidal et al. reported the most common cause was lymphoid neoplasia (38.9 % of patients) [31]. wAIHA secondary to drugs was observed in a single case in the seven studies (allopurinol) [5].

“Two studies reported symptoms at the time of presentation; jaundice was the most prevalent symptom in both, occurring in 73.0 % and 78.7 % of patients.” [5,32].

The laboratory results of Hb, indirect bilirubin, LDH, and reticulocytes reported are summarized in Table 2. Hb was considerably reduced among patients with wAIHA, with the mean/median values falling below the cut-off (<8 g/dL) thereby defining severe anemia in five of the six studies with available information [35]. Conversely, hemolytic markers (indirect bilirubin, LDH, and reticulocytes) were generally elevated.

**Table 2:** Summary of laboratory data at patient presentation.

Author, Year	Hemoglobin, g/dL	Indirect bilirubin, mg/dL	LDH, U/L	Reticulocyte, %
Jaime-Pérez et al. 2019 [14]	6.7 (1.0–10.8)	1.7 (0.4–9.8)	434 (4–2938)	8.2 (0.0–65.0)
López-Vidal et al. 2019 [31]	5.8	Not reported	449	Not reported
Hernandez-Company et al. 2017 [5]	6.4 (2.0–14.0)	2.18 (0.2–9.7)	437 (140–4124)	5.5 (0.5–20.0)
Agramonte et al. 2015 [32]	6.9 (4.2–10.0)	0.3 (0.2–0.4)	890 (450–1120)	Not reported
Quintero 2015 [30]	9.1 (*n* = 70)	2.5 (*n* = 50)	Not reported^a^	3.5 (*n* = 43)
Hernández et al. 2013 [34]	6.9 g/L (2.8–10.2)	Not reported	Not reported	13.4 (4.0–24.0)

LDH, lactate dehydrogenase.

Results are presented as median (minimum-maximum) except for López-Vidal et al. [31] and Hernández et al. [5] – mean (minimum-maximum).

a Results on LDH were provided qualitatively: 52 patients (73.2 %) had elevated LDH and data were missing for the remaining 14 patients.

### Response and remission criteria

Six studies evaluated response rates to wAIHA treatments. Of these, four detailed the criteria considered to define treatment response (Table 3). The two studies evaluating disease remission rates specified the definition used.

**Table 3:** Criteria used to define treatment response and disease remission.

Author, Year	Definitions
**Treatment response definitions**	
Jaime-Pérez et al. 2019 [14]	CR: Hb ≥12 g/dL in women and ≥13 g/dL in men PR: Hb ≥10 g/dL with persistent hemolysis or with a ≥ 2.0 g/dL increment in basal Hb at diagnosis without reaching normal values
Hernandez-Company et al. 2017 [5]	CR: Hb >12 g/dL and a normalization of all hemolysis markers PR: Hb >10 g/dL or an increment of 2 g/dL over the basal Hb without the need for transfusions
Quintero 2015 [30]	CR: Hb >11 g/dL in primary wAIHA and normal Hb levels according to age and sex in secondary wAIHA with negative hemolysis markers and a negative DAT PR: Hb >9 g/dL without the need for transfusions
Hernández et al. 2013 [34]	Treatment response to corticosteroid treatment: patients who received one to two cycles of prednisone at a dose of 60 mg/day for 3 months and who had the dose gradually decreased while maintaining hemoglobin and reticulocyte counts within normal values without treatment or with maintenance doses of 5–15 mg per day
**Disease remission definitions**	
López-Vidal et al. 2019 [31]	Complete remission: Hb ≥12 g/dL without signs of hemolysis (normal reticulocytes, bilirubin, and lactate dehydrogenase) or need for treatment Partial remission: Hb ≥12 g/dL and need to maintain corticosteroid therapy
Hernández et al. 2013 [34]	Disease remission: patients with Hb levels and reticulocyte count within normal values, with a negative DAT, and without immunosuppressive treatment for at least one year

CR: complete response; PR: partial response; Hb: hemoglobin; DAT: direct antigen test.

The lack of a current consensus on the definitions of complete and partial response to wAIHA treatments is evident in Table 3. Though the definitions used by Jaime-Pérez et al. [14] and Hernandez-Company et al. [5] were similar, slight differences are still observed. For instance, the latter required the normalization of all hemolysis markers and made no distinction between sexes regarding the Hb target (>12 g/dL) in the definition of complete response (CR), and considered that patients receiving blood transfusions were not partial responders.

### Treatment

#### Corticosteroids

Corticosteroids were used for the first-line treatment of nearly all patients enrolled in the studies (346 out of 349 patients - 99.1 %) with available data. The chosen corticosteroid (prednisone, methylprednisolone, or dexamethasone), posology, and corresponding treatment outcome are summarized in Table 4.

**Table 4:** Summary of first-line treatments and outcomes.

Author, Year	First-line treatment	Number of patients	Drug and posology	Outcomes	Median (min-max) time to response
Jaime-Pérez et al. 2019 [14]	Corticosteroids	46	Prednisone (1–2 mg/kg/day) for 28 days	Response rate: 78.0 % CR: 35 % PR: 43 %	11.5 days (1–30 days)
			Dexamethasone (40 mg/day) for 4 days		
	Rituximab plus corticosteroids	18	Rituximab (100 mg/week) plus high-dose dexamethasone for 4 weeks	Response rate: 100 % CR: 11.1 % PR: 88.9 %	14 days (3–30 days)
López-Vidal et al. 2019 [31]	Corticosteroids	36	Prednisone (1 mg/kg/day) for at least 3 weeks	Remission rate: 92.0 %	Not reported
			Dexamethasone (40 mg/day) for 4 days		
			Methylprednisolone (500 mg/day) for 3 days		
Hernandez-Company et al. 2017 [5]	Corticosteroids	89	Methylprednisolone (1 g/day) for 3 days followed by prednisone (1 mg/kg/day) for 30 days	Response rate: 84.3 % CR: 50.6 % PR: 33.7 %	27 days (9–44 days)
Agramonte et al. 2015 [32]	Corticosteroids^a^	15	Not reported	Response rate: 73.3 % CR: 33.3 % PR: 40.0 %	Not reported
Quintero 2015^b^ [30]	Corticosteroids	52	Not reported	Response rate (*n* = 40): 90.0 %^c^ CR: 16.7 % PR: 83.3 %	Not reported
	Combined corticosteroid therapy (10 with azathioprine and 1 with an alkylating agent)	11	Not reported		
	Other	3	Not reported		
Hernández et al. 2013 [34]	Corticosteroids	44	Prednisone (one or two cycles with a 60 mg/day dose) for 3 months	Response rate: 77.3 %^d^ Remission rate: 75.0 %	Not reported
Valdés et al. 2009 [33]	Corticosteroids	35	Not reported	Response rate: 68.6 %^e^	Notreported

Min: minimum; max: maximum; CR: complete response; PR: partial response.

a Two patients required the administration of intravenous immunoglobulin.

b Information on first-line treatment was missing for five patients.

c Response rate was reported for 40 of the 71 patients (56.3 %) included in the study and corresponds to the overall response observed across first-line treatments.

d Five of the patients (14.2 %) responded after a second cycle of prednisone.

e Response rate was reported for the total of 35 patients included in the study, two of whom had cold AIHA.

The great majority (90.8 %) of the 349 patients received corticosteroids alone as the first-line treatment for wAIHA. Response rates ranged from 68.6–84.3 % in the included studies. The timing of treatment response assessments was specified by two studies at three and four weeks after starting treatment in the studies by Hernandez-Company et al. [5] and Agramonte et al. [32], respectively. Two studies computed median times to response: 11.5 days (range: 1–30 days) [14] and 27 days (range: 9–44 days) [5]. The proportion of patients attaining disease remission with first-line corticosteroid treatment was 75.0 % and 92.0 % in the studies that assessed this outcome [31,34]. Long-term outcomes were evaluated by two studies. The median duration of response, defined as the time from response to relapse or death, was 22 months (range: 11–31 months) in the study by Jaime-Pérez et al. [14]. In addition, the median relapse-free survival was 81.7 months in this study [14] and 11.7 months for primary wAIHA and 6.6 months for secondary wAIHA as reported by Hernandez-Company et al. [5]. The former study defined relapse as a reemergence of disease, while the latter considered it a decrease in the Hb level or the appearance of hemolytic markers after patient response.

Combined corticosteroid therapy was used as the first-line treatment in 29 of the 349 patients (8.3 %). In the study by Jaime-Pérez et al. [14], 18 patients received low-dose rituximab (100 mg/week for four weeks) plus high-dose dexamethasone (HDD) as front-line therapy for wAIHA at a reference center in Mexico. Of these 18 patients, two (11.1 %) attained a CR and 16 (88.9 %) achieved a PR, with a median time to response of 14 days (range: 3–30 days). The median duration of response was 16.5 months (range: 1–39 months). No statistically significant differences in relapse-free survival were observed between patients treated with low-dose rituximab plus HDD and those treated with corticosteroids alone at the same center. In a study conducted in medical institutions in Bogota, Colombia, treatment outcomes were not available for ten patients treated with corticosteroids plus azathioprine and one patient with corticosteroids plus an alkylating agent [30].

Corticosteroid use beyond the first-line treatment was reported by only one study, where four relapsing patients received a new cycle of corticosteroids in the second line, resulting in a 100 % remission rate [31].

#### Splenectomy

Splenectomy was not used for the treatment of wAIHA in two studies. In a study conducted at Hospital del Salvador, Chile, none of the 36 patients diagnosed with wAIHA between 2010 and 2018 underwent this surgical procedure [31]. The authors based their decision on the fact that splenectomy is not as effective in secondary wAIHA, which accounted for most patients at the center. Similarly, in a study conducted in Cuba, none of the four patients requiring second-line treatment underwent splenectomy [32].

In contrast, splenectomy was the only second-line treatment used in a referral center in Mexico City and the most common second-line treatment in another study conducted in Cuba (Table 5) [5,34]. In the former study, 36 patients with wAIHA (corresponding to 40.4 % of all those enrolled) underwent splenectomy, 61.1 % of whom experienced a CR and 33.3 % a PR. Although splenectomy was deemed safe and effective, study authors underlined that this procedure should be deferred in patients with secondary wAIHA in light of the associated risks of developing infectious or thrombotic complications [5]. In the study conducted in Cuba [34], seven of ten patients (70.0 %) who required second-line treatment underwent splenectomy, corresponding to 15.9 % of included patients. The disease remission rate was 30.0 %. In five of these seven patients, other treatments were administered in addition to splenectomy including intravenous immunoglobulin (*n* = 3), azathioprine (Imuran), cyclophosphamide (*n* = 2), danazol (*n* = 1), and vincristine (*n* = 1).

**Table 5:** Summary of treatments and outcomes in refractory wAIHA.

Author, Year	Treatment	Number of patients	Treatment line	Outcome
Hernandez-Company et al. 2017 [5]	Splenectomy	36	Second	Response rate: 94.4 % CR: 61.1 % PR: 33.3 %
Hernández et al. 2013 [34]	Splenectomy^a^	7	Second	Remission rate: 30.0 %
Valdés et al. 2009 [33]	Splenectomy	4	Second	Response rate: 100 %
López-Vidal et al. 2019 [31]	Corticosteroids	4	Second	Remission rate: 100 %
López-Vidal et al. 2019 [31]	Rituximab	2	Second	Remission rate: 100 %
López-Vidal et al. 2019 [31]	Immunosuppressants • Cyclophosphamide (*n* = 1) • azathioprine (Imuran)	2	Second	Remission rate: 100 %
Valdés et al. 2009 [33]	Immunosuppressants • Cyclophosphamide (*n* = 1) • azathioprine (Imuran)	7	Second	Response rate: 100 %
Hernández et al. 2013 [34]	Immunosuppressants (azathioprine)	1	Second	Remission: No
	Cyclophosphamide, vincristine, and intravenous immunoglobulin	1	Second	Remission: Yes
	Intravenous immunoglobulin	1	Second	Remission: Yes
Quintero 2015 [30]	Immunosuppressants (azathioprine)	16	Second	Response rate (*n* = 16): 75.0 %^b^ CR: 31.3 % PR: 43.7 %
	Splenectomy	4	Second	
	Alkylating agents	2	Second	
	Danazol	2	Second	
	Intravenous immunoglobulin	2	Second	
	Other	2	Second	
Hernandez-Company et al. 2017 [5]	Rituximab	2	Third	Not reported
Hernandez-Company et al. 2017 [5]	Immunosuppressants (azathioprine, cyclophosphamide, and vincristine)	30	Third	Response rate: 93.7 %^c^ CR: 43.7 % PR: 50.0 %
Jaime-Pérez et al. 2019 [14]	Splenectomy	7	Second to fourth	Response rate: 100 %^f^
Jaime-Pérez et al. 2019 [14]	Immunosuppressants (cyclophosphamide)	5	Second to fourth	Not reported
Jaime-Pérez et al. 2019 [14]	Intravenous immunoglobulin	2	Second to fourth	Not reported
Jaime-Pérez et al. 2019 [14]	Danazol	2	Second to fourth	Not reported
Jaime-Pérez et al. 2019 [14]	Rituximab (100 mg/week) plus high-dose dexamethasone for 4 weeks	8	Third to fifth^d^	Response rate: 100 % CR: 37.5 % PR: 62.5 %
Jaime-Pérez et al. 2019 [14]	Immunosuppressants (cyclophosphamide)	5	Third to fifth^d^	Transient response followed by relapse
Jaime-Pérez et al. 2019 [14]	Low dose rituximab	5	Fourth to sixth^e^	Response rate: 100 %

CR: complete response; PR: partial response.

a In five patients, other treatments were administered in addition to splenectomy: intravenous immunoglobulin (*n* = 3), azathioprine (Imuran), cyclophosphamide (*n* = 2), danazol (*n* = 1), and vincristine (*n* = 1).

b Response rate was reported for 16 of the 28 patients (57.1 %) who received second-line treatments and corresponds to the overall response observed in the second line.

c The rate was computed for a total of 32 who required third-line treatment at the site – two of whom received low dose rituximab plus high-dose dexamethasone.

d These patients were part of a subgroup of ten patients with refractory disease who had received a median of 2.5 (2–4) lines of treatment, including steroids (100 %), intravenous immunoglobulin (20 %), cyclophosphamide (50 %), danazol (20 %), and splenectomy (70 %).

e Patients who relapsed after cyclophosphamide received low dose rituximab.

f Followed by relapse.

Use of splenectomy for wAIHA treatment was also observed in the remaining three studies, primarily in the second-line therapy. In a study conducted in Cuba, 4 of 11 patients (36.4 %) who did not successfully respond to first-line corticosteroid treatment underwent splenectomy as the second-line therapy (response rate of 100 %) [33]. Four of 28 patients (14.3 %) who required second-line treatment for wAIHA underwent splenectomy in a study conducted in Colombia [30]. In a study conducted at a reference center in Mexico, seven patients (10.9 % of the study sample) underwent splenectomy, either as their second-, third-, or fourth-line treatment. Though a 100 % response rate was obtained, all patients subsequentially relapsed. The authors of this study reported that most patients declined to undergo splenectomy [14].

#### Rituximab

Data on the use of rituximab for the treatment of wAIHA are scarce in Latin America. Excluding first-line treatment, a total of 17 patients received rituximab in these studies (Table 5). Two patients were treated with rituximab as second-line therapy in the study by López-Vidal et al. [31] (remission rate of 100 %) and another two received this monoclonal antibody as third-line therapy in the study by Hernandez-Company et al. [5] (treatment outcome not reported). Low-dose rituximab (100 mg/week) for four weeks plus HDD was administered to eight patients refractory to several lines of therapy (range: 2–4) in the study by Jaime-Pérez et al. [14]. CR and PR rates were 37.5 % and 62.5 %, respectively with the median time to response being 16 days. The response was maintained for an average of 81.9 months. At the same center, five refractory patients who relapsed following treatment with cyclophosphamide (in the third to fifth lines) successfully responded to low-dose rituximab, with a median response maintenance of 100 months.

#### Immunosuppressants

All six studies that characterized treatments beyond the first line recorded the use of immunosuppressants for wAIHA management. Overall, 67 patients received immunosuppressants in the included studies, primarily as second- and third-line therapies (over 85.1 % of patients) (Table 5). Of the 30 patients treated with immunosuppressants as the third-line therapy, 43.7 % experienced a CR and 50.0 % a PR. Of note, this rate was computed for a total of 32 patients who required third-line treatment at the center, two of whom received low-dose rituximab plus HDD.

#### Other treatments

Treatments other than corticosteroids, splenectomy, rituximab, and immunosuppressants were rarely administered, occurring in only 15 patients across all included studies (Table 5). These included intravenous immunoglobulin (nine patients), danazol (four patients), and alkylating agents (two patients).

Three studies evaluated transfusion requirements among patients with wAIHA, with the proportion of patients receiving blood transfusions during the follow-up varying between 19.1 % and 39.4 % [5,14,30].

### Safety data

Whereas all seven studies evaluated the effectiveness of wAIHA treatments, safety data were reported by only one study. Jaime-Pérez et al. [14] reported that no adverse effect was observed among the 18 patients who received low-dose rituximab plus HDD as first-line therapy for wAIHA.

### Mortality

Two studies reported mortality data. López-Vidal et al. [31] found a mortality rate of 20.9 % in a sample of 43 patients with AIHA (36 with wAIHA [83.7 %] and the remaining seven with cold AIHA [16.3 %]) followed up for an average of 38 months. None of the patients died due to the anemia but as a result of the secondary causes: gastric cancer (*n* = 3), chronic lymphocytic leukemia (*n* = 3), and non-Hodgkin lymphoma (*n* = 1). No cause of death was reported for two of the patients. Jaime-Pérez et al. [14] reported mortality results exclusively for wAIHA patients. In this study, two out of 64 patients with wAIHA (3.1 %) died during long-term follow-up. One death was secondary to Kaposi sarcoma in an HIV-positive patient while the other was a result of an ischemic stroke associated with a hemolytic episode.

### Unmet needs

In the study by Hernández et al. [34], rituximab was not available at the center during the analyzed period (1997–2006). Financial barriers further limit access to rituximab, even in centers where this drug is available. In a referral center in Mexico City, only two out of 32 patients who required a third-line treatment were able to receive rituximab due to economic constraints [5]. Importantly, this was the study where the second-line treatment was comprised exclusively of splenectomy. Though not stated by the authors, the lack of other treatment options owing to financial barriers might have contributed to the frequent use of splenectomy at the center. Additionally, in a different center in Mexico, authors reported that low-dose rituximab was administered due to financial restrictions in the low-income, uninsured patient population treated at the center [14].

### Clinical practice guidelines and healthcare policy documents

The literature search retrieved a single national clinical guidelines for AIHA issued in Mexico [36]. In addition, a Clinical Protocol and Therapeutic Guidelines (CPTG) on AIHA was issued in 2018 by the Ministry of Health in Brazil [17]. This document, intended primarily as a healthcare policy aimed at guiding clinical-decision making and resource allocation within the public health system, does not constitute a national clinical guidelines.

Both the Mexican guidelines and the Brazilian CPTG provide guidance on the diagnosis and treatment of AIHA, including specific recommendations for wAIHA. Both documents recommend corticosteroids, namely prednisone and methylprednisolone, as the first-line treatment for wAIHA. Additionally, both include splenectomy as a second-line treatment for patients who either do not respond to corticosteroids or require high maintenance doses to control the disease. However, while splenectomy is the sole second-line treatment presented in the Mexican guideline, the Brazilian CPTG further states that immunosuppressants (cyclophosphamide or cyclosporine) can also be administered for patients refractory to corticosteroids. In the third line, the Brazilian CPTG details that immunosuppressants may be used in patients refractory to splenectomy. The Mexican guidelines provide several treatment options for patients with Hb levels below 10 g/dL after splenectomy. These include rituximab, immunosuppressants (cyclophosphamide, cyclosporine, azathioprine, and mycophenolate mofetil), and alemtuzumab. Immunoglobulin is recommended in both documents only in select cases, namely in severe patients with Hb below 7 g/dL in Brazil, and in those with cardiac or pulmonary disease who require transfusions and in refractory chronic patients in Mexico. The Brazilian CPTG also establishes recommendations on the monitoring and post-treatment follow-up of AIHA patients.

## Discussion

The present scoping review aimed to evaluate and summarize the available evidence reported on adult patients with wAIHA in Latin America. A thorough literature search was performed, encompassing various databases and gray literature. Therefore, we are confident that we have identified the majority of available literature on wAIHA in Latin America.

As is the case outside Latin America, studies on wAIHA are lacking and the scarce evidence available is primarily derived from retrospective single-center case series of patients.

As expected, the results show that women are disproportionally affected by wAIHA. This is likely explained by the fact that women have an increased susceptibility to autoimmune diseases [5], and is aligned with published literature [23,26]. The present review also confirms that systemic lupus erythematosus is a frequent underlying cause of wAIHA, being the most common in five of six studies that included patients with secondary wAIHA. Literature data show that AIHA occurs in 5–10 % of patients with systemic lupus erythematosus [37].

Corticosteroids are the mainstay first-line treatment for wAIHA, being recommended as such in both the Mexican guidelines and the Brazilian CPTG [17,36]. Accordingly, all but three of the 349 patients with non-missing information enrolled in the included studies received corticosteroids as the first-line treatment, in the great majority of cases as monotherapy (90.8 %). Prednisone, methylprednisolone, and dexamethasone were used.

Response rates to first-line treatments based on corticosteroids alone ranged from 68.6–84.3 % in these studies. Differences in response rates between studies might partly result from the variability in doses and durations of corticosteroid treatment and definitions of treatment response. These findings align with published literature reporting that between 70–80 % of patients generally respond to corticosteroid treatment [23,24]. The attainment of a CR to first-line corticosteroid treatment is particularly important, as this is associated with long-term remission. In a study by Roumier et al. [24], long-term remission was recorded in 92 % and 53 % of patients with a CR and a PR to first-line corticosteroids, respectively. Moreover, patients with PR may still present mild to moderate anemia, as the cut-off points defined for Hb in the response criteria were >9 g/dL and ≥10 g/dL in the three included studies that specified this information [5,14,30]. The CR rates observed in the studies included in this review (33.3–50.6 %) are also consistent with published literature [24,26]. The median relapse-free survival after first-line corticosteroid treatment differed significantly between the two studies that reported this outcome: 11.7 and 6.6 months in primary and secondary wAIHA [5], respectively, versus 81.7 months in a study where approximately half of the patients had secondary wAIHA [14]. This discrepancy may be, at least in part, a result of differences in relapse definitions, study populations, and type and duration of first-line corticosteroid treatment between the studies.

Though response rates to corticosteroid treatment were high, one should note that a considerable proportion of patients required second-line therapies (15.6–40.4 %). These findings converge with those reported by other studies, which showed that the rate of long-sustained remission after tapering and discontinuation of first-line corticosteroids is low (30–40 % after one year) [22,38,39]. Additionally, other literature found proportions of patients requiring second-line treatments after an initial response to corticosteroids (28.3 % and 52.2 %) similar to those in the present review [24,26]. Information on corticosteroid dependency was scarce in the included studies. Published data show that a high proportion of corticosteroid responders become corticosteroid dependent. In a study conducted in France, the rate of corticosteroid dependency among patients initially responding to corticosteroid treatment was above 60 % [24]. In another study of 44 patients receiving first-line prednisone, nine (20.5 %) required long-term maintenance immunosuppression. Of these nine individuals, seven also required the introduction of second-line agents [34].

While the first-line treatment of wAIHA was based almost exclusively on corticosteroids, the second line was characterized by various treatment options. Second-line treatment of wAIHA has classically been based on splenectomy, the effectiveness of which is similar to that of rituximab while providing slightly longer remissions [7]. Nevertheless, reports outside Latin America have described a gradual decline in the use of this invasive procedure, which is currently employed in <10 % of cases [7,40]. This decline has mostly been attributed to the resulting increase in infection risk and thrombotic complications. Moreover, splenectomy entails some additional downsides, namely the lack of reliable predictors of outcome and other potential surgical complications [7,40]. We found different practices regarding the use of splenectomy as the second-line treatment of wAIHA in Latin American centers. These practices ranged from a complete absence of splenectomies in two of the studies (in Chile [31] and Cuba [32]) to splenectomy being the sole second-line treatment in a referral center in Mexico City [5].

European data have demonstrated an emergence of rituximab as the preferred second-line option in the last decades [24]. Our findings show that rituximab was used in only two out of the 93 patients (2.2 %) who received second-line treatments in the included studies. Moreover, due to the limited number of studies and countries contributing data, these results may not accurately reflect the current use of rituximab in the management of wAIHA throughout Latin America. One of the possible reasons behind the limited use of this monoclonal antibody is the fact that rituximab is not currently approved for the treatment of wAIHA. Reasons reported by the retrieved studies included the unavailability of rituximab at some centers, as well as economic constraints limiting its use for the treatment of wAIHA [5,34].

The healthcare burden among relapsing patients is significant and underestimated [3]. These patients have a higher transfusion burden and a higher rate of alloimmunization [41]. Currently, the treatment of patients with relapsing disease and unfit for or refusing splenectomy constitutes an important clinical challenge [42,43]. Treatment options for such patients are few (namely azathioprine, cyclophosphamide, and cyclosporine) and of limited efficacy. Novel agents currently being studied in wAIHA, such as bortezomib, nipocalimab, rilzabrutinib, and ianalumab, might become important future treatment options for patients with relapsing disease [[42], [43], [44], [45], [46]].

The present literature review shows that several data gaps and challenges related to wAIHA currently exist in Latin America. First, epidemiological and healthcare resource utilization data are scarce, and no data were retrieved on patient-related outcomes, quality of life, or perspectives on wAIHA and its daily burden. In addition, whereas all seven studies evaluated the effectiveness of treatments given to wAIHA patients, only one reported safety results, specifically evaluating the short- to long-term safety of low-dose rituximab plus HDD as first-line therapy. Furthermore, data on blood transfusions among these patients were provided by only three of the studies and were limited to the proportion of those receiving transfusions during follow-up. Additional details, such as the number of transfusions per patient, patient response to transfusion, or the setting in which the transfusion was performed (inpatient versus outpatient), which would be essential to fully characterize transfusion burden in wAIHA patients, were not reported. Second, all studies determined response and/or remission rates as a means of evaluating the effectiveness of wAIHA treatments. However, other important treatment-related outcomes, such as time to response and relapse-free survival, have seldom been reported. Third, there are currently no standardized criteria to evaluate response (complete or partial) to wAIHA treatments, as is the case internationally [47,48]. The variability in the definitions used impairs the validity of cross-study comparisons. Lastly, information on patients refractory to wAIHA treatment, particularly to third line therapies and beyond, is scarce. However, the data reported by Jaime-Pérez suggest that an appreciable proportion of wAIHA patients (over 15 %) require more than two lines of treatment [14]. Further research is warranted to understand the management strategies and unmet needs of patients who fail to control the disease after several lines of treatment.

This study is not without limitations. Only four Latin American countries were represented in the present literature review, with studies conducted in Cuba accounting for 42.9 % of those included. As a result, our findings might not be representative of the reality throughout Latin America. In addition, most studies were small case series (four included <50 patients), and all were retrospective.

Though the population of interest in this review were adult patients with wAIHA, we decided to include studies that did not report data exclusively for patients ≥18 years or with the wAIHA subtype. Three of the studies, Quintero [30], Jaime-Pérez et al. [14], and Hernandez-Company et al. [5], presented data for samples of wAIHA patients aged ≥15, ≥16, and ≥17 years, respectively. In the study by Valdés et al. [33], two of the 35 enrolled patients had cold AIHA. The decision to not exclude these studies from the review was based on the scarcity of available data on wAIHA patients in Latin America. The impact of this limitation is expected to be minimal, as the study samples were still comprised almost exclusively of adult patients with wAIHA.

As previously shown, the definitions of CR and PR varied. Moreover, the timing of the assessment of treatment response differed between both studies that provided this information: three and four weeks after the start of first-line treatment with corticosteroids in the studies by Hernandez-Company et al. [5] and Agramonte et al. [32], respectively. The full extent of this limitation is difficult to ascertain as information on the timing of treatment response assessment was not available in the remaining studies. Both these factors might influence response rates (e.g., more stringent response criteria and earlier assessment of response to treatment may both lead to lower response rates) and should thus be taken into account when comparing the results of these studies.

The treatment outcome rates (response or remission) reported by the individual studies, particularly for treatments beyond the first line, should be interpreted cautiously considering the small number of treated patients.

### Conclusion

The present review summarized the available evidence on adult patients with wAIHA generated in Latin America. Studies on wAIHA are lacking and the scarce evidence available is primarily derived from retrospective case series of patients conducted at single centers. Corticosteroids were part of the first-line treatment of wAIHA in virtually all patients enrolled in these studies, mainly as monotherapy (91 %). Response rates were high (69–84 %), but a considerable proportion of patients required second-line treatments (15–40 %). Treatment approaches beyond first-line therapies varied significantly between countries and institutions, likely due to a lack of disease-specific therapeutic options, the absence of standardized guidelines throughout Latin America, and barriers to access newer therapies. Data on patients refractory to several lines of treatment are currently very limited.

## Funding

This work was supported by Johnson & Johnson Innovative Medicine (LATAM region). The sponsor was involved in the study design, collection and interpretation of data, writing of the report, and in the decision to submit the article for publication.

## Author contribution

Yudy Medina, Renato Watanabe de Oliveira, and Pamella Villanova developed the protocol outline and search strategy. Yudy Medina and Renato Watanabe de Oliveira conducted the literature search, which was reviewed by Diogo Ribeiro. Yudy Medina and Diogo Ribeiro performed the data extraction. Diogo Ribeiro drafted the manuscript draft, and all other co-authors contributed to writing, reviewing, and editing. All authors read and approved the final manuscript.

## Data availability

The data that support the findings of this study are available from the corresponding author upon reasonable request.

## Conflicts of interest

Juan Jiménez and Sandra Gualandro report no conflicts of interest. Yudy Medina, Renato Watanabe de Oliveira, and Pamella Villanova are employees of Johnson & Johnson, the study sponsor. Diogo Ribeiro is an employee of CTI Clinical Trial & Consulting, which is a consultant to Johnson & Johnson, the study sponsor.

## Appendix Supplementary materials

Image, application 1
