# Supplementary material for: Warm autoimmune hemolytic anemia in adults in Latin America: A scoping review
Source: Hematol Transfus Cell Ther. 2026 Mar 8;48(2):106351. doi: 10.1016/j.htct.2026.106351 (PMC12991941; doi:10.1016/j.htct.2026.106351)
Supplement: Supplementary file 1 [file mmc1.docx]

**Supplementary Table 1:** Search strategy

| **Databases** | **Search Strategy** |
| --- | --- |
| *Medline and Embase* |  |
| 1. | exp Anemia, Hemolytic, Autoimmune/ or warm autoimmune hemolytic anemia.mp. or waiha.mp. |
| 2. | exp Latin America/ or exp Colombia/ or exp Brazil/ or brasil.mp. or exp Mexico/ or exp Argentina/ or exp Panama/ or exp Chile/ or latin america.mp. or exp guatemala/ or exp costa rica/ or exp el salvador/ or exp haiti/ or republica dominicana.mp. or exp bahamas/ or exp central america/ |
| 3. | 1 AND 2 |
|  |  |
| *Cochrane Library* |  |
| 1. | Anemia, Hemolytic, Autoimmune |
| *LILACS* |  |
| 1. | (Anemia, Hemolytic, Autoimmune) OR (warm autoimmune hemolytic anemia) OR waiha |
| *Value in Health* |  |
| 1. | (Anemia, Hemolytic, Autoimmune) OR (warm autoimmune hemolytic anemia) |
| *Epistemonikos* |  |
| 1. | Anemia, Hemolytic, Autoimmune OR warm autoimmune hemolytic anemia OR waiha |
| 2. | (waiha) AND (latin america OR latin OR colombia OR brazil OR mexico OR argentina OR panama OR peru OR ecuador OR latam OR costa rica OR chile OR caribbean) |
| 3. | 1 AND 2 |
| *Grey literature (Google)* |  |
|  | (Anemia, Hemolytic, Autoimmune) OR (warm autoimmune hemolytic anemia) OR waiha AND (Brasil OR México OR Latinoamérica OR Colombia) |
